# *En Bloc* Tumor Resection, Optical Molecular Imaging, and the Potential Synergy of the Combination of the Two Techniques in Bladder Cancer

**DOI:** 10.3389/fonc.2021.638083

**Published:** 2021-03-16

**Authors:** Yongjun Yang, Chao Liu, Xiaoting Yan, Jiawei Li, Xiaofeng Yang

**Affiliations:** ^1^ First Clinical Medical College, Shanxi Medical University, Taiyuan, China; ^2^ Department of Urology, First Hospital of Shanxi Medical University, Taiyuan, China

**Keywords:** bladder cancer, optical molecular imaging, *en bloc* tumor resection, detection, surgery

## Abstract

Although transurethral resection of bladder tumor is the golden standard for the treatment of non-muscle invasive bladder cancer, this surgical procedure still has some serious drawbacks. For example, piecemeal resection of tumor tissue results in exfoliated tumor cells dissemination and implantation, and fragmented tumor specimens make it difficult for pathologists to accurately assess the pathological stage and histologic grade. En bloc tumor resection follows the basic principle of oncological surgery and provides an intact tumor specimen containing detrusor muscle for pathologists to make accurate histopathological assessment. However, there is no robust clinical evidence that en bloc tumor resection is superior to conventional resection in terms of oncological outcomes. Considering the high recurrence rate, small or occult tumor lesions may be overlooked and incomplete tumor resection may occur during white light cystoscopy-assisted transurethral resection. Molecular fluorescent tracers have the ability to bind tumor cells with high sensitivity and specificity. Optical molecular imaging mediated by it can detect small or occult malignant lesions while minimizing the occurrence of false-positive results. Meanwhile, optical molecular imaging can provide dynamic and real-time image guidance in the surgical procedure, which helps urologists to accurately determine the boundary and depth of tumor invasion, so as to perform complete and high-quality transurethral tumor resection. Integrating the advantages of these two technologies, optical molecular imaging-assisted en bloc tumor resection shows the potential to improve the positive detection rate of small or occult tumor lesions and the quality of transurethral resection, resulting in high recurrence-free and progression-free survival rates.

## Introduction

Bladder cancer (BC) is the tenth most common cancer disease worldwide with 474 000 new incident cases and 197 000 deaths annually, and it is also the second most common malignant disease of the urinary system after prostate cancer (1). About 75% of newly diagnosed BC cases present as a lesion confined to the mucosa or submucosa, collectively referred to as non-muscle invasive bladder cancer (NMIBC) (2). For these patients, transurethral resection of bladder tumor (TURBT) combined with personal intravesical chemotherapy or immunotherapy that is tailored to tumor risk stratification is recommended as the routine treatment model by the major international guidelines (2–5). The quality of transurethral tumor resection plays an important role in histopathological assessment and treatment decision-making, which affects the prognosis of the disease. However, conventional TURBT has some serious drawbacks. First, piecemeal resection of tumor tissue leads to the dissemination and implantation of exfoliated tumor cells, which goes against the recognized principle of oncological surgery and contributes to increases in the rate of tumor recurrence (6–8). Second, fragmented tumor specimens make it difficult for pathologists to accurately assess the pathological stage and histologic grade (9). Finally, small or occult malignant lesions, particularly carcinoma *in situ* (CIS), are not easy to visualize and diagnose in the bladder wall during white light cystoscopy (WLC)-assisted transurethral resection (10). For NMIBC patients, accurate histopathological evaluation, especially of the boundary and depth of tumor invasion, is essential for selecting an optimal treatment strategy (11). Furthermore, the quality of the initial transurethral resection strongly determines overall medical cost of BC treatment (12). Therefore, there is an urgent need to improve the surgical procedure of transurethral tumor resection to solve the serious drawbacks of WLC-assisted conventional resection.

In the past decade, en bloc resection of bladder tumor (ERBT) has served as a valuable alternative technique that has attracted increasing interest among urologists globally (13). Compared with conventional resection, ERBT has three potential advantages with respect to tumor cell implantation, perioperative complications, and specimen quality. First, the technique follows the principle of cancer surgery and removes the tumor in one piece, thereby minimizing the number of exfoliated tumor cells and reducing the risk of cell implantation. Second, the more precise and controllable procedure of tumor resection may reduce the incidence of perioperative complications, such as blood loss, obturator nerve reflex (ONR), and bladder perforation (BP). Finally, an intact tumor specimen containing detrusor muscle (DM) can be collected after en bloc resection for accurate histopathological assessment by pathologists (14).

Optical molecular imaging is a novel molecular targeted imaging technology that can realize qualitative and quantitative analysis of pathological processes at the cellular and molecular levels prior to macrostructure changes of malignant tissue (15). The molecular fluorescent tracer specifically binds to the tumor site and then highlights the malignant lesions from normal mucosa tissue under a paired optical imaging device. Due to its high sensitivity and specificity, the positive detection rate of small or occult tumor lesions, especially CIS, can be improved (16–18). Meanwhile, optical molecular imaging can provide dynamic and real-time images during transurethral tumor resection, which is helpful for urologists to accurately determine the boundary and depth of tumor invasion.

Theoretically, integrating the advantages of the above two technologies, patients who receive optical molecular imaging-assisted en bloc resection may achieve high-quality and complete tumor resection. In this review, we focus on the evidence for the use of ERBT and optical molecular imaging in BC, and point out their potential clinical value in future applications.

## Clinical Benefits of EN Bloc Resection of Bladder Tumor

The well-known principle of oncological surgery is to remove the tumor tissue in one piece with negative surgical margin and prevent iatrogenic tumor cells scattering and local implantation. Therefore, if a surgeon cuts the renal cell carcinoma tissue into pieces and disperses it throughout the surface of the remaining normal renal tissue when performing nephron-sparing surgery, he or she will be punished or even dismissed. However, for NMIBC, urologists do this every day during conventional resection without any restrictions. ERBT adheres to the principle of cancer surgery and provides pathologists with an intact tumor specimen containing DM (19–21). Meanwhile, through more controllable and precise surgical procedures of transurethral resection, the risk of perioperative complications in ERBT, such as blood loss, ONR, and BP, is reduced compared with conventional TURBT. As for oncological outcomes, there is still no robust clinical evidence that patients with NMIBC can benefit from ERBT (13, 14).

## Histopathological Evaluation

The choice of treatment strategy for NMIBC depends on the clinicopathological characteristics of tumor lesions, such as stage, grade, number, diameter, prior recurrence rate, and coexistence of CIS (2). Tumor specimens collected after surgery should be sufficient for pathologists to evaluate the clinicopathological characteristics and perform risk stratification of tumor tissue (22). Conventional TURBT ignores the principle of oncological surgery and removes tumor tissue piece by piece. Moreover, thermal damage, electrocautery artifacts and lack of spatial orientation of fragmented tumor specimens increase the difficulty of accurately assessing the pathological stage and histologic grade (9). The incidence of tumor upstaging found at repeat transurethral resection (reTUR) was 0%–8% (Ta to ≥T1) and 0%–32% (T1 to ≥T2) in patients with BC (23). Meanwhile, in a large retrospective cohort study including 18 277 patients diagnosed with T1 high-grade BC during the initial tumor resection, 41% of patients had tumor upstaging and 12.7% had positive nodes in the final histopathological analysis of specimens obtained from radical cystectomy (RC) (24).

The presence or absence of DM in tumor specimens is the most important marker for the quality and completeness of transurethral tumor resection (25). A high proportion of DM presence was found in the intact tumor specimens collected after en bloc resection (**Tables 1** and **2**). Bipolar electrodes have better hemostatic effects than monopolar electrodes. Therefore, under the guidance of clear operative vision, the surgical procedure of en bloc resection with a bipolar electrode as an energy source can be performed more controllably and precisely, making sure that the tumor specimens contain lamina propria and superficial muscle layers with minimal crush and cautery artifacts (38, 39). A European multicenter study was conducted to compare the safety and efficacy of ERBT using different energy sources. The results showed that the rate of DM presence in tumor specimens was high, and it was similar in electrical ERBT (96%) and laser ERBT (100%) (41).

**Table 1:** Studies comparing perioperative complications, detrusor muscle, and oncological outcomes between transurethral resection of bladder tumor (TURBT) and *en bloc* resection of bladder tumor (ERBT) categorized on their level of evidence (LoE).

Study	Operation	Energy source	Patients	ONR	BP	Transfusion	Bladder irritation	Blood loss	Urethral stricture	DM	Follow-up	Recurrence	Progression
Randomized comparing studies (LoE 1b)													
Zhang et al. (26)	ERBT TURBT	Thulium laser Bipolar	149 143	N.A.	0* 6	N.A.	N.A.	N.A.	N.A.	131^†^ 134	At 36 mo	REC: 45.6%^†^ 42.7%	5.4% ^†^ 7.7%
Chen et al. (27)	ERBT TURBT	Thulium laser Monopolar	71 71	0* 18	0^†^ 0	N.A.	N.A.	17.2ml^†^ 14.5ml	N.A.	N.A.	At 18 mo	REC: 5.6%^†^ 9.9%	0^†^ 0
Non-randomized comparing studies (LoE 2a–2b)													
Bălan et al. (8)	ERBT TURBT	Bipolar Monopolar	45 45	2* 5	N.A.	N.A.	N.A.	0.28 g/dL* 0.76 g/dL	N.A.	N.A.	At 12 mo	REC: 17.1%* 27.5%	N.A
Hayashida et al. (19)	ERBT TURBT	Polypectomy snare Monopolar	39 31	N.A.	N.A.	0^†^ 0	N.A.	N.A.	N.A.	39 N.A.	Mean 12 mo	REC: 15.4%^†^ 19.4%	N.A.
Yang et al. (28)	ERBT TURBT	Monopolar Monopolar	96 87	23^†^ 21	2* 9	N.A.	N.A.	N.A.	N.A.	39^†^ 66	At 24 mo	REC: 21.2%^†^ 27.5%	N.A.
Zhang et al. (29)	ERBT TURBT	Monopolar Monopolar	40 50	9^†^ 12	2^†^ 4	N.A.	N.A.	N.A.	N.A.	40* 27	Median 10.8 mo Median 11.3 mo	REC: 20.0%^†^ 24.0%	N.A.
Tao et al. (30)	ERBT TURBT	980nm laser Monopolar	36 48	0* 6	0* 3	0^†^ 0	N.A.	N.A.	N.A.	N.A.	At 12 mo	REC: 2.8%^†^ 4.2%	N.A.
Cheng et al. (31)	ERBT TURBT	KTP laser Monopolar	34 30	0* 10	N.A.	N.A.	N.A.	5.0^†^ 7.5	N.A.	33* 24	At 12 mo	REC: 8.8%* 33.3%	0.0%* 16.7%
Li et al. (32)	ERBT TURBT	Thulium laser Bipolar	136 120	0* 4	0^†^ 1	N.A.	N.A.	N.A.	N.A.	130* 103	Mean 41 mo Mean 40.6 mo	RFS: 31.8%^†^ 27.9%	N.A.
D’souza et al. (33)	ERBT TURBT	Holmium laser Monopolar	23 27	0* 11	0* 3	N.A.	5* 14	N.A.	2^†^ 2	N.A.	At 36 mo	REC: 30.4%^†^ 37.0%	N.A.
Chen et al. (34)	ERBT TURBT	Green laser Monopolar	83 75	0* 9	0^†^ 2	N.A.	N.A.	0.87g/ml* 1.00g/ml	1^†^ 3	N.A.	At 36 mo	N.A.	N.A.
Xu et al. (35)	ERBT TURBT	Thulium laser Monopolar	26 44	0* 7	0^†^ 3	0^†^ 0	N.A.	N.A.	N.A.	N.A.	12 to 24mo	REC: 15.4%^†^ 27.3%	N.A.
Migliariet al. (36)	ERBT TURBT	Thulium laser Monopolar	58 61	0* 8	0^†^ 3	0^†^ 2	N.A.	N.A.	N.A.	58* 53	Mean 20 mo	REC: 20.6%^†^ N.A.	0 N.A.
Cheng et al. (37)	ERBT TURBT	HybridKnife Monopolar	95 98	2^†^ 7	0^†^ 2	N.A.	N.A.	2.76 g/L^†^ 3.00 g/L	0 0	N.A.	1 to 33mo	RFS: 95.5%* 79.5%	N.A.

ONR, obturator nerve reflex; BP, bladder perforation; DM, detrusor muscle; N.A., not applicable; KTP, potassium-titanyl-phosphate; REC, recurrence rate; RFS, recurrence-free survival.

*Compared to TURBT, the rate was significantly decreased.

^†^Compared to TURBT, the rate was not significantly decreased.

**Table 2:** Studies analyzing perioperative complications, detrusor muscle, and oncological outcomes of *en bloc* resection of bladder tumor (ERBT).

Study	Energy	Patients	ONR	BP	Transfusion	Bladder irritation	Blood loss	Bladder bleeding	Urethral stricture	DM	Follow-up	Recurrence	Progression
Zhang et al. (38)	Bipolar	82	0	0	N.A.	N.A.	N.A.	0	N.A.	100%	At 18 mo	RFS: Ta: 88.5% T1: 74.5%	N.A.
Abotaleb et al. (39)	Bipolar	46	0	0	1	0	1.3g/dL	3	N.A.	100%	At 12 mo	REC: 15.2%	N.A.
Zhang et al. (20)	Vela laser	38	0	0	0	0	N.A.	0	0	100%	At 12 mo	REC: 21.6%	N.A.
Hurle et al. (40)	Monopolar	74	N.A.	1	N.A.	N.A.	N.A.	0	N.A.	100%	At 24 mo	REC: 17.6%	N.A.
Xu et al. (21)	Thulium laser	141	N.A.	2	N.A.	N.A.	N.A.	N.A.	N.A.	100%	At 36 mo	RFS: 68.3%	0

ONR, obturator nerve reflex; BP, bladder perforation; DM, detrusor muscle; N.A., not applicable; RFS, recurrence-free survival; REC, recurrence rate.

In a recent meta-analysis, T1 substaging was shown to be closely related to the oncological outcomes of patients with NMIBC (42). A total of 601 patients with T1 BC were retrospectively followed up for 5.9 years. The results indicated that metric substaging was the best independent prognostic indicator of progression-free and cancer-specific survival rates (43). The substaging of pT1 BC depends on the depth of tumor invasion in muscularis mucosae (MM). Compared with conventional TURBT, the anatomical architecture and spatial orientation of tumor specimens are well preserved during ERBT, which helps to minimize inter-observer variability when pathologists identify pT1 BC substaging (44). Moreover, the spatial orientation of the biopsy sample is helpful in distinguishing MM from DM, thus allowing to accurately stage pT1 versus pT2 disease (45, 46). Lymphovascular invasion (LVI) is another important prognostic factor for the recurrence and progression of NMIBC (47). Unfortunately, no studies have explored whether en bloc resection is beneficial for diagnosis of LVI. According to the International Collaboration on Cancer Reporting (ICCR) guidelines, in addition to pathological stage and histologic grade, status of muscularis propria, histological variant and LVI should be included in pathology reports of tumor specimens obtained from biopsy or transurethral resection as required items, while T1 substaging is listed among the recommended items (48). However, it is almost impossible to collect all tumor fragments during conventional resection, so urologists prefer to select some representative samples and submit them to the pathology department. An intact and high-quality tumor specimen can be obtained through ERBT, which helps pathologists to make detailed pathological reports. Now, close cooperation and comprehensive information sharing between urologists and pathologists are advocated to comprehensively evaluate the clinicopathological characteristics and accurately stratify the risk of tumor tissue, and then formulate an optimal treatment strategy for NMIBC patients (49).

## Perioperative Complications

During conventional TURBT, the tumor tissue is resected piece by piece from an exophytic part of the tumor to the superficial muscular layers by a wire loop. From the perspective of equipment, monopolar energy generates high-frequency currents that flow through the resectoscope to the grounding pad adhered to the patient’s lower limb, resulting in ONR and related BP due to muscle contraction and thermal damage to adjacent tissues. Moreover, the use of electrolyte-free solutions as intraoperative irrigation fluid increases the risk of TUR syndrome (50).

The general principle of ERBT is to make a circular mucosal incision at a safe distance from the tumor base and then remove the whole tumor tissue including superficial DM (51, 52). So far, the safety and efficacy of en bloc resection have been explored in several clinical trials, and the results showed that the perioperative complication rate was not higher than that of conventional resection (**Table 1**). The monopolar current originally used in conventional TURBT can also be applied as an energy source for en bloc resection. Although the occurrence of ONR is similar to that of conventional resection, BP can be controlled within an acceptable range through elaborate surgical procedures and less frequent cutting and coagulation (28, 29, 40). With the introduction of laser energy into en bloc resection, ONR could be avoided and BP occurred in only two patients in a series of studies involving 795 patients (20, 21, 26, 27, 30–36). However, the occurrence of ONR and BP was 12.0%–40.7% and 0%–11.1% in conventional TURBT, respectively (26, 27, 31–36). For the single-arm studies about en bloc resection, the incidence of perioperative complications was negligible (**Table 2**).

## Can *EN Bloc* Resection Improve Oncological Outcomes?

In conventional resection, the integrity of tumor tissue is destroyed and tumor cells are dispersed, which increases the risk of dissemination and implantation of exfoliated tumor cells. Meanwhile, due to continuous bladder irrigation, the pressure in the bladder is higher than the venous pressure, causing exfoliated tumor cells to travel into the blood circulation *via* the vascular system (53, 54). En bloc resection, as a “no touch” technique for the treatment of NMIBC, shows the potential to minimize the number of exfoliated tumor cells and reduce the risk of tumor cells entering the blood circulation (55). Theoretically, ERBT can achieve the envisaged goal of decreasing the rate of tumor recurrence and progression.

**Table 1** lists the studies comparing the oncological outcomes of TURBT and ERBT, and **Table 2** lists the results of single-arm studies of en bloc resection. As shown in **Table 1**, there was a general downward trend in tumor recurrence and progression of the ERBT group. However, the data of oncological outcomes in clinical trials presented large variability across centers or urologists (30, 31), and not all studies have confirmed that en bloc resection was superior to conventional resection (26, 27). Meanwhile, different energy sources and no standardized postoperative intravesical instillation therapy have been used in different studies, resulting in non-comparable data on tumor recurrence and progression. In short, there is no robust clinical evidence that ERBT is superior to conventional resection with respect to oncological outcomes (13).

## Avoiding Repeat Transurethral Resection

According to the European Association of Urology (EAU) guidelines, reTUR is recommended for patients with incomplete initial tumor resection, no DM in Ta high-grade specimens, and all T1 tumors (2). The reTUR has two goals: to detect and remove residual tumors, and to reconfirm the pathological stage. However, reTUR is an invasive surgical procedure that may seriously affect the quality of patients’ life and increase their negative emotions (56). Moreover, elderly patients with other serious diseases cannot tolerate this procedure under spinal or general anesthesia. Finally, the implementation of reTUR will increase overall health care costs and the economic burden of individual patients. Therefore, without compromising oncological outcomes, the selection of the right patients to avoid reTUR is particularly important.

A retrospective multicenter study collected 321 patients with T1 high-grade bladder tumor who underwent reTUR. The results showed that the presence of DM in the initial tumor specimens, the absence of concurrent CIS, and en bloc tumor resection were three independent predictors of the absence of residual tumor at reTUR (57). After thulium laser en bloc resection, the short-term oncological outcomes of recurrence and progression were not significantly different between patients who received reTUR and those who did not (58). With the use of new optical imaging technologies in clinical practice as adjunct to WLC, such as photodynamic diagnosis (PDD) and narrow-band imaging (NBI), the detection rate of BC has been improved (59). Based on this theoretical evidence, the new optical imaging technology-assisted ERBT is expected to achieve optimal initial transurethral resection and then reduce the need for reTUR in well-selected patients with NMIBC (60).

## Application of Optical Molecular Imaging in the Management of Bladder Cancer

After WLC-assisted TURBT, around 15%–61% and 31%–78% of patients with NMIBC will develop tumor recurrence within one and five years, respectively (2). The high recurrence rate may be attributed to tumor multicentricity, incomplete tumor resection, implantation of exfoliated tumor cells, and neogenetic tumor formation. WLC, a traditional optical imaging system for the diagnosis and treatment of suspected BC, has several drawbacks. First, CIS is a high-risk kind of NMIBC confined to the mucosa, which can easily be confused with inflammatory lesions due to its similar structural appearance under WLC. A recent systematic review was conducted to analyze the results of random bladder biopsy in >10 000 patients with NMIBC, and the total incidence of CIS was 17.35% (61). Moreover, the rate of concurrent CIS reach up to 50% in patients with high-grade or sessile tumors (61, 62). Second, small or occult tumors, some of which may be high-grade or invasive lesions, are difficulty to detect (63). Finally, the boundary and depth of tumor invasion judged by the two-dimensional images of cystoscopy and operator’s clinical experience are often subjective and inaccurate, even among senior urologists (64). Imprecise estimation of tumor tissue depth and demarcation impedes the completeness of tumor resection. Although no solid data have indicated that ERBT is superior to conventional TURBT with respect to oncological outcomes, it should be noted that 77% of tumor recurrence was located away from the original surgical site after en bloc resection (65), while most residual tumors (36%-86%) found at reTUR were located at the initial resection site after conventional resection (23). Thus, in order to detect more small or occult tumor lesions, especially CIS, during transurethral resection, new adjunctive optical imaging technologies for WLC are urgently needed.

According to the scope of imaging field, optical imaging technologies can be grossly divided into two groups: macroscopic and microscopic models. Macroscopic imaging modalities, such as PDD and NBI, provide a wide field of view of bladder wall in a similar manner to WLC and rely on additional contrast enhancement to highlight uncertain malignant lesions (37). Compared with WLC-assisted TURBT, the blood loss, ONR, and BP are significantly reduced, and the recurrence-free survival rate is significantly improved in patients treated with NBI-assisted en bloc resection (66). Even though PDD and NBI have the ability to improve the detection rate of papillary lesions and CIS, the incidence of false-positive results in prior intravesical therapy, inflammation, and intraoperative acute hemorrhage was significantly increased due to non-tumor specificity (67). Optical coherence tomography (OCT) and confocal laser endomicroscopy (CLE) belong to microscopic imaging technologies that produce high-resolution images of abnormal bladder mucosa to provide real-time pathological information about the changes in tissue microstructure and cell morphology (68, 69). However, OCT and CLE can only provide a limited view of bladder tissue during the examination. These technologies need to be combined with another macroscopic imaging modality (such as WLC, PDD, or NBI) to delimit the boundaries of tumor tissue (70).

Optical molecular imaging is a new molecular-targeted imaging technology that can qualitatively and quantitatively analyze the pathological process at the cellular and molecular levels prior to macrostructural changes of malignant tissue (15). The urinary bladder is a highly compliant hollow organ that can provide perfect closed operating dark environment for optical molecular imaging without interference from external stray light sources. Moreover, intravesical instillation of fluorescent targeted tracers through the urethra is convenient and simple before surgery. Molecular targeted tracers have the ability to bind tumor cells with high sensitivity and specificity. Optical molecular imaging mediated by it can detect small or occult tumor lesions while minimizing the occurrence of false-positive results.

## Bladder Cancer Detection

CD47, a member of the immunoglobulin superfamily, is overexpressed on more than 80% of BC cell membranes but not on normal urinary tract epithelium (71). When CD47 binds to signal regulatory protein α on phagocytes, it can inhibit the phagocytosis of tumor cells by macrophages to promote tumor proliferation (72). After RC, twenty-one fresh intact bladder specimens were collected and incubated with anti-CD47-Qdot625, and then detected under blue light. Finally, a total of 119 suspicious bladder tissues were examined, and the sensitivity and specificity were 82.9% and 90.5%, respectively (16). Carbonic anhydrase IX (CAIX), as a member of the carbonic anhydrase family, participates in intracellular pH modulation under hypoxic conditions, thus changing the biological features of tumor in terms of proliferation, adhesion, and progression (73). Using a similar experimental strategy, after incubating with anti-CAIX-Qdot625, the entire mucosa of the fresh intact bladder specimen was carefully examined under WLC, and then detected under blue light cystoscopy. The overall sensitivity and specificity for BC detection under WLC were 76.0% and 90.5%, while a high detection accuracy was achieved and the sensitivity and specificity rose to 88.00% and 93.75% under CAIX targeted optical molecular imaging (17).

Unlike monoclonal antibodies, peptides are synthesized by phage-display peptide libraries or the one-bead one-compound combinatorial chemistry approach, with the smallest molecular weight (0.5–2.0 kDa) among molecular tracers (74). The CSNRDARRC peptide, which specifically binds to human bladder tumor HT-1376 cells, is the first targeting peptide selected by phage-display libraries. In the N-butyl-N-(4-hydroxybutyl) nitrosamine-induced BC model, the fluorescein-conjugated CSNRDARRC peptide actively targets the luminal epithelium of malignant lesions, but not to normal bladder regions after intravesical instillation of bladder (75). The CSSPIGRHC peptide (NYZL1), which binds to human bladder tumor BIU-87 cells, was also selected by phage-display technology. After intravenous administration of fluorescein-labeled NYZL1 peptide, the tracer specifically binds to malignant tissue in a nude mouse model of human bladder tumor xenografts (76). The CSDRIMRGC peptide is another bladder tumor-specific peptide, named PLSWT7. A preclinical trial was conducted to explored the diagnostic accuracy of the corresponding molecular fluorescent tracer in the diagnosis of malignant lesions in eight fresh intact bladder specimens. The specimens were incubated with PLSWT7-IRDye800CW, and the sensitivity and specificity of optical molecular imaging-assisted BC detection were 84.0% and 86.7%, respectively (77). Unlike the previous peptides, the CQDGRMGFC peptide (PLZ4) is synthesized by the combinatorial chemical method and can specifically bind to bladder tumor cells. An *in vivo* research showed that after intravenous administration, PLZ4-Cy5.5 selectively labeled the tumor tissue in the patient-derived xenograft mouse model (78).

Tumor heterogeneity can occur between different patients, or even in the same patient, which greatly impairs the diagnostic accuracy of molecular targeted tracers mediated optical molecular imaging (79). Many researchers have found that malignant cells, including BC cells, have increased glycolytic activity, resulting in an acidic tumor microenvironment (80, 81). The pH low insertion peptides (pHLIPs) target the acidity on the surface of tumor cells, and then penetrate the cancer cell membrane. The acidic tumor microenvironment contributes to this pathological process (82, 83). Twenty-two fresh intact bladder specimens were incubated with indocyanine green (ICG)-labeled pHLIP, and then the bladder mucosa was examined using paired optical imaging equipment. Regardless of the pathological stage, the sensitivity and specificity of malignant tissue detection were 97% and 100%. However, when including necrotic and previously treated tissues with intravesical chemotherapy, the total number of false-positive results increased and the specificity decreased to 80% (18).

A variety of molecular fluorescent agents have been explored in optical molecular imaging of bladder tumor and have shown the ability to improve the detection rate of BC lesions in urothelial cancer animal models and patient’s tumor specimens. Tumor heterogeneity and different protein expression patterns between patients complicate the selection of molecular fluorescent tracers (84). Then the following question arises: How can urologists choose the best molecular targeted agent to perform optical molecular imaging-assisted en bloc resection for a specific patient with NMIBC. Fortunately, according to the EAU guidelines, cystoscopy-guided biopsy is strongly recommended for patients with suspected malignant lesions, followed by histopathological evaluation of the tissue samples as the initial diagnostic procedure (2). Immunohistochemical analysis and next-generation sequencing of tumor tissue can reveal biomarkers for molecular imaging, thereby helping urologists choose the most appropriate molecular fluorescent tracer for candidates who may benefit from optical molecular imaging (85).

## Real-Time Guidance in Bladder Cancer Surgery

During WLC-assisted TURBT, urologists rely on their own clinical experience and indirect visual feedback to determine the location of the tumor lesion and the boundary and depth of tumor invasion. Unfortunately, at the time of reTUR, residual tumors occur in 51% of patients with T1 bladder tumors, which reflects the inaccuracy of initial intraoperative evaluation (2). For NMIBC patients, complete resection of all visible malignant lesions and accurate histopathological evaluation of tumor specimens are the keys to prolonging recurrence-free and progression-free survival rates. Meanwhile, in order to avoid unnecessary deepening and expanding resection to preserve more adjacent normal tissues, urologists need extra intraoperative optical imaging guidance to perform complete and high-quality tumor resection, regardless of the surgeon’s clinical experience in urological endoscopic surgery. Optical molecular imaging is a promising adjunctive imaging mode for WLC in BC surgery. In a preclinical trial, bladder tumor xenograft mouse models were randomly subdivided into two groups, and tumor resection was operated under the sunlight condition (control group, *n* = 20) and with optical molecular imaging guidance (experimental group, *n* = 20). A week later, the recurrence rates of the control group and experimental group were 95% and 5%. At 30 days after operation, the overall survival rates of the two groups were 0% and 90%, respectively (77).

**Figure 1:**
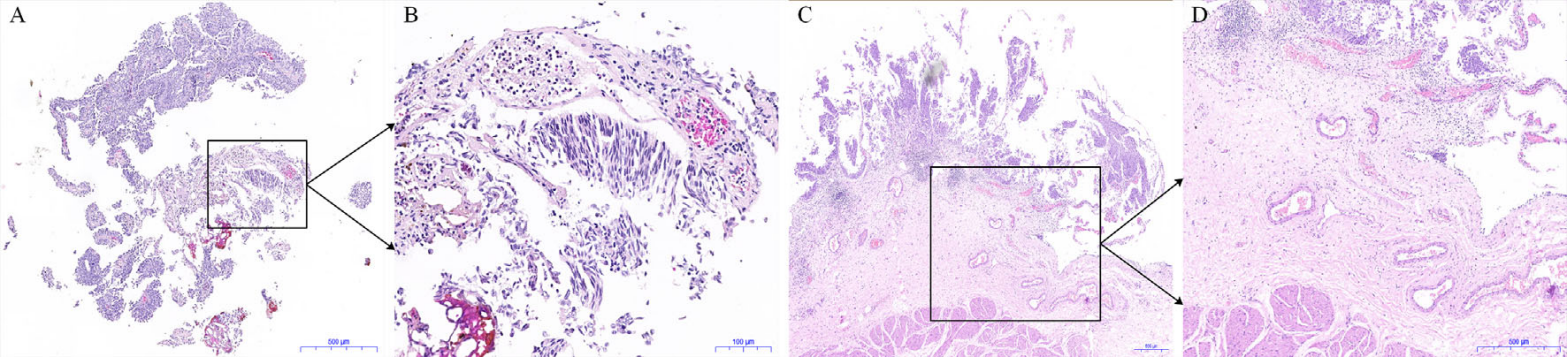
Histological pictures of the tumor specimens obtained from conventional transurethral resection of bladder tumor (TURBT) and en bloc resection of bladder tumor (ERBT). **(A)** The hematoxylin and eosin (HE) staining image of tumor specimen obtained from conventional TURBT. **(B)** The enlarged image in the black frame in **(A)**. **(C)** The HE staining image of tumor specimen obtained from ERBT. **(D)** The enlarged image in the black frame **(C)**.

**Figure 2:**
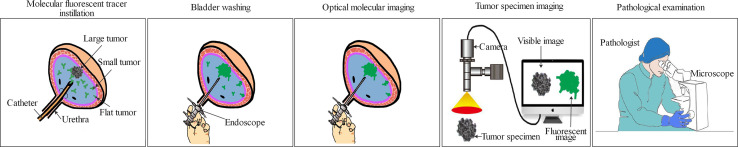
Principle of optical molecular imaging-assisted en bloc resection for the treatment of bladder cancer. After instillation of the molecular targeted tracer (from half an hour to a few days before operation relying on the pharmacokinetics and distribution of the molecular targeted tracer in vivo) into the bladder, the bladder is flushed to drain the uncombined molecular targeted tracer. Then, a paired imaging device is used for optical molecular imaging to detect the entire bladder mucosa. In addition to large tumors, small or occult tumor lesions can be detected under optical molecular imaging-assisted en bloc resection. The intact tumor specimens collected after the operation is immediately sent to the pathology department and examined using fluorescence microscope. Pathologists can accurately assess the status of the tumor surgical margin and the depth of tumor invasion, which will help urologist choose the most appropriate treatment strategy for patients with non-muscle invasive bladder cancer to improve the oncological outcomes.

The fresh intact bladder tumor specimens collected after ERBT can help pathologists make accurate histopathological assessments and assess the status of tumor surgical margin. After the intact tumor specimen was incubated with anti-CD47-Alexa Fluor 790 and imaged under a near-infrared (NIR) imaging device, the mean fluorescence intensity of tumor tissue was significantly higher compared with adjacent normal background tissue, which might further assist pathologists in selecting the best pathological material to find the positive surgical margin (86). Therefore, on the one hand, en bloc tumor resection can ensure accurate pathological evaluation of the resected specimens through better protecting the spatial orientation and architecture of the tumor tissues during surgery (87). For another, optical molecular imaging shows the potential to improve the positive detection rate of small or occult tumor lesions. Meanwhile, based on the difference in the fluorescence signal between tumor tissue and adjacent normal tissue, urologists can objectively judge the depth and boundary of tumor invasion during transurethral resection. Therefore, optical molecular imaging-assisted en bloc tumor resection could help urologists perform high-quality and complete tumor resection. After surgery, an intact tumor specimen containing DM can be collected for pathologists for histopathological assessment (**Figure 1**). Then the risk stratification of NMIBC and the depth of tumor invasion can be assessed accurately and objectively. Based on the above information, urologists can select the most appropriate treatment strategy for patients with NMIBC to improve the oncological outcomes (**Figure 2**).

## Challenges and Future Perspectives

The major challenge in performing ERBT is the size of bladder tumor (13). In the ERBT group, most clinical trials excluded patients with tumor diameter larger than 3 cm (8, 19, 31). Although a previous study reported that HybridKnife-assisted en bloc resection is suitable for larger bladder tumors with diameters up to 7.5 cm, the intact tumor specimen retrieval remains a significant challenge (88). Without the help of additional surgical instruments, it is impossible to extract a large intact tumor specimen *via* the outer sheath of the resectoscope (89). Given the high medical costs of NMIBC (90), some medical equipment manufacturers have designed new medical devices to improve the quality of surgery. Meanwhile, urologists should work closely with equipment manufacturers to improve resection instruments and extraction devices to ensure that transurethral tumor resection follows the basic principle of oncological surgery. In addition, all patients have a strong desire to receive high-quality tumor resection in order to avoid further reTUR, especially among the high-risk NMIBC patients (91).

Optical molecular imaging is a new and promising visualization system for tumor diseases. However, the available evidence is limited to preclinical trials. There are some technical and scientific issues that urgently need to be resolved before patients and urologists can reap the benefits of high diagnostic accuracy associated with it. For instance, to achieve a balance between drug safety and fluorescence image quality, it is essential to carry out dose optimization experiments. Meanwhile, the use of bright fluorescent targeted molecules and sensitive optical imaging equipments guarantees high-resolution images. Up to now, the only NIR imaging device approved for clinical application by the US Food and Drug Administration is designed and developed based on the photophysical properties of ICG (92). However, for some newly synthesized fluorophores, due to their unique photophysical characteristics, they might not be well compatible with the existing imaging devices, so a customized optical imaging equipment may be required to present high-resolution images. Compared with WLC, extra optical imaging device and molecular targeted tracers are required to perform optical molecular imaging. With respect to the cost-effectiveness of this new optical imaging technology, the costs of fluorescent targeted molecules, amortization of imaging devices, and the benefits obtained from the improvement of oncological outcomes should be taken into account. Similarly, PDD and NBI, as enhanced imaging systems, have been recommended by the EAU guidelines for transurethral tumor resection, which also requires extra optical imaging devices (2). Due to the reduced recurrence rate and the associated reoperation rate, PDD-assisted TURBT can save €168 per patient per year (93), and obtain the greatest economic benefits among moderate-risk NMIBC patients (94). For patients with NMIBC receiving NBI-assisted TURBT, compared with WLC-assisted TURBT, each patient can save $230 to $500 per year (95).

Molecular targeted tracers and optical imaging equipments are two basic essentials for optical molecular imaging. However, there is no expert consensus on the standardized evaluation of the performance of molecular targeted tracers, which makes it difficult to organize prospective multicenter studies and analyze the experimental data from different scientific research institutions. The fluorescence intensity of the tissue to be examined and the tumor-to-background ratio (TBR) are two important references for evaluating the clinical application value of molecular targeted tracers. However, the fluorescence signal of the detection area is affected not only by the drug dosage, pharmacokinetics, and the duration between administration of molecular targeted tracers and imaging examination, but also by the manufacturer, technical parameters, and performance of the optical imaging device used (96). In addition, even if a satisfactory TBR could be observed during the inspection, the data collection might be affected by the region of interest selected by different operators.

The ultimate goal of related research is to introduce optical molecular imaging technology into the clinical application of en bloc tumor resection. Therefore, a promising fluorescent targeted tracer for molecular imaging must pass the drug security analysis before being explored in clinical studies. But unfortunately, the drug toxicity analysis and good production process are expensive and time-consuming, which makes them the major obstacles to the design and development of new fluorophores and molecular tracers. Ideally, molecular targeted tracers should have the following characteristics, such as good drug safety, high tumor tissue specificity, appropriate pharmacokinetics and chemical stability in the human body.

## Conclusions

ERBT is a safe and feasible technique for the treatment of NMIBC, and it provides an intact tumor specimen containing DM for pathologists for accurate histopathological assessment. Indeed, there is no robust clinical evidence that ERBT performs better than conventional resection in terms of oncological outcomes. Optical molecular imaging shows the potential to improve the detection rate of malignant lesions and highlight the boundary and depth of tumor invasion. In theory, optical molecular imaging-assisted en bloc tumor resection integrates the advantages of both and is expected to improve the quality and completeness of transurethral tumor resection, and ultimately improve the oncological outcomes of patients with NMIBC.

## Author Contributions

YY designed the study, collected and analyzed clinical data, and wrote the manuscript. CL, XtY, and JL collected the related literature. XfY designed the study, supervised the research, and reviewed the manuscript. All authors contributed to the article and approved the submitted version.

## Funding

The research is supported by the National Natural Science Foundation of China (NSFC, Grant No. 81172444).

## Conflict of Interest

The authors declare that the research was conducted in the absence of any commercial or financial relationships that could be construed as a potential conflict of interest.

## Abbreviations

BC, Bladder cancer; NMIBC, non-muscle invasive bladder cancer; TURBT, transurethral resection of bladder tumor; CIS, carcinoma in situ; WLC, white light cystoscopy; ERBT, en bloc resection of bladder tumor; ONR, obturator nerve reflex; BP, bladder perforation; DM, detrusor muscle; reTUR, repeat transurethral resection; RC, radical cystectomy; MM, muscularis mucosae; LVI, lymphovascular invasion; ICCR, International Collaboration on Cancer Reporting; EAU, European Association of Urology; PDD, photodynamic diagnosis; NBI, narrow-band imaging; CLE, Confocal laser endomicroscopy; OCT, optical coherence tomography; CAIX, Carbonic anhydrase IX; pHLIPs, pH low insertion peptides; ICG, indocyanine green; NIR, near-infrared; TBR, tumor-to-background ratio.
